# The New Common

**DOI:** 10.1007/s10978-021-09293-z

**Published:** 2021-05-16

**Authors:** Stephanie Jones

**Affiliations:** Department of English, University of Southampton, Southampton, SO17 1BJ UK

**Keywords:** Animals, Commons, Dasgupta review, Nature, Southampton

## Abstract

This article describes a personal relationship to a common green space in a town in the United Kingdom during the lockdowns prompted by the Covid-19 pandemic in 2020–21. It considers the new meanings that are attaching to ‘commons’ as conceptual spaces and material places; and it links this consideration to the terms in which values and norms are being reassessed in the context of environmental crises.

## Background

I stand as close to the tree as I can get, as still as I can be. A few metres away, over a low wall and across the unusually quiet road, people queue under make-shift flood lights. They are masked, of course, and carefully spaced. It is not so cold but most wear coats. They shuffle forwards as directed by marshals wearing Hi Vis gilets. Inappropriately, my mind connects to scenes from books and movies about totalitarian regimes.

I am playing hide and seek with my younger daughter and looking at the transformation of the Atherley Bowling Club into a Covid testing site. A new variant has been identified in our area: surge testing provides the background to our game. We are playing in a patch of trees within the crowded nineteenth-century cemetery on the south western corner of Southampton Common. I am wary for two reasons. First, because I need to judge the right time to make a noise or a movement so I can be found with excitement rather than anxiety. If I hide too well for too long, my daughter will fear I have disappeared, become lost or been taken. The other is because of the proximity of the gravestones. I worry that it might seem disrespectful or be sacrilegious to hide and seek surrounded by the dead, albeit the long dead. What is graveyard etiquette? What is graveyard etiquette now?

## Foreground

On the straight and narrow yew-lined path that runs through the ‘new’ (but still old) cemetery leading into the open fields of The Common, people step ostentatiously aside to lend two metres for others to pass. It is often impossible not to step onto a grave site, but this seems okay, although people hold themselves with care, often looking up and away. Or rather they aim their gaze to ensure they are exhaling away, newly self-conscious of breath as a multiplicity of invisibilities. These pauses between the trees can provide an opportunity to lock eyes with one of The Common’s infamously bold rats; or to wonder at an angel with muscular wings but no nose or hands, broken wrists held in glory. Or to imagine being killed by lightning as a teenager, or to wonder what it would be like to live to an old age, beyond the early deaths of seven of your children. Beyond the path, a variety of huge old evergreen trees create a vista of varied textures and gnarly shapes. Late in February, these stepping-asides start to involve another new etiquette that entails looking assiduously down rather than performatively up and away, as we all try not to tread on opening-closing crocuses or the wax perfect tilt of snowdrops.

But as February turns into March, I am almost annoyed that so many people are walking, running, and cycling around The Common. These 365 acres of woodland, field, marsh and pond are a minute’s walk from our backyard, and the place we can go when we cannot go anywhere. With milder days, it has become critically crowded again, as it did during the first lockdown last March. I have contradictory responses. I feel proprietorial, although I have only come to know The Common intimately in the past year, having lived by it for a decade. This is my daily space, and these people are surely trespassers. But I second-guess this selfish instinct: I am, after all, part of this carefully distanced flocking, this wary expression of the fundamental human instinct for community life. In Wikipedia’s words, ‘it has been suggested that the area’s status as a common goes back to the town of Hamwic around 500 AD’ (Southampton Common 2021). It has been a site of cow-herding, water-sourcing, brick-making, beer-brewing, horse-racing, zoo-keeping, and festival-making across the centuries. It has also been a juridical site beyond a history of legal wrangling over public-right versus private-use of the land itself: if you know where to look at the northerly end of the common, you can imagine the traditional meeting mound of the Court Leet. And last summer, my older daughter and I were thrilled to find the Tate Modern prominently displaying a work of feminist and animal rights performance art-activism recording the interruption of The Southampton Horse Show (English 2013[1975]). To find our near-backyard featured in a major art gallery made us feel somehow glamourous. These days, the spectacle of prancing horses and caged tigers has been replaced by the mostly abstract knowledge that The Common is one of few abodes of the Great Crested Newt. Although after heavy rain a couple of years ago, we found one of these rarely seen creatures washed onto the path, helplessly displaying its vibrant orange belly marked by cleanly defined black splotches, its stubby limbs splayed and viscous. The angling of its wide-apart eyes made it seem anxious, although its wider mouth lent it a sanguine expression. Maybe nervous-calm is not an oxymoron for a newt. We put it in a rivulet and felt like saviours, momentarily. It floated oddly, only one limb stiffly motioning through silty water.

## Middleground

In the UK, it has become a cliché that the lockdowns prompted by the pandemic are bringing us closer to nature and nature closer to us. We are apparently more attuned to the seasons than ‘before’, despite all the time indoors. A surprisingly bright ochre and shockingly glossy fox—a fairytale creature—now casually and proprietorially traverses a path between our place and The Common. Quieter streets have allowed us to hear birdsong and indeed bird noise: I am always amazed by the speed and volume of the wood-peckers’ tree hammering that resonates from The Common. (Google takes me to measurements of wood-pecking decibels higher than the colossal sounds of containers being loaded and unloaded at the Southampton Docks a couple of miles down the road.) While bodies of birds—individual shapes, or in sweeping flocks— are traditional harbingers of change across many cultures, the sound of birdsong reassures us of a world continuing its established patterns. One of the many legacies of Rachel Carson’s foundational text of environmentalism, *Silent Spring*, is that the absence of birdsong has now become the more ominous modern harbinger of change than any swooping visitation (1962).

‘Our economies, livelihoods and wellbeing all depend on our most precious asset: Nature’. This is one of the Headline Messages of *The Economics of Biodiversity: The Dasgupta Review*. ‘We are all asset managers’. But to describe Nature as an ‘asset’ seems at odds with the other central rhetoric of the review. ‘We—and our economies—are embedded within nature, not external to it’ (Dasgupta 2021). On the one hand, the review encourages individuals, communities, and institutions to lend definitive monetary value to Nature so that loss can take on harder meaning within the market, driving innovation and investment. On the other hand, the review encourages us to apprehend ourselves as ‘embedded’ in nature, which is surely to imagine ourselves unbound from the market, delimited from monetarised value. The Review, in other words, involves a rhetorically certain sense of capitalist *fait accompli* and a rhetorically less certain anti-consumerist impetus. The authors seek a middle ground within the long running and widely staged debate about whether capitalism and a sustainable future are or are not compatible. From a position of critique, then, the Dasgupta Review seems to lead to aporia. Nature needs to be framed as an asset because it is not even or only an asset… The end of history is still the end of history, even as we realise the urgent need to make new history… While the review presses for a radical reassessment of values, it does not promise new norms. And whether new values are enough or new norms are necessary is the ‘old chestnut’ of environmental debate. Middle grounds are slippery and can even be treacherous.

Last autumn, the ground of The Common was slippery with crushed chestnuts and their decomposing casings: strangers would remark to one another on the remarkable ‘crop’. I do not know if this was an abundance to be celebrated or a marker of nature going awry, a sign of unequal times, a last huzzah, an inadvertent symbol of the decline of some other species. The unusual behaviours of the natural world compel us and make us nervous. This year as last year, our milder climate has brought the growth of the chestnut catkins forward to March, a timing that could be but cannot be explained by Southampton’s naturally warmer micro-climate. But it is difficult to sustain unease as The Common is so lovely, such a relief in the spring.

As a matter of critique, engaging with ‘the commons’ increasingly involves thinking about the in-common. The bright aesthetics of shared resource management—golden wheat, milk-heavy cows—has given way to broader inter-species approaches. And the less edifying ‘tragedy of the commons’—exhausted crops, overgrazed fields—has given way to a more expansive sense of—in Donna Haraway’s words—‘staying with the trouble’ of ‘fellow critters’, our ‘kin’. This year I have been teaching ecocriticism, aquatic environmentalism and animal poetics. Taking these classes online has been particularly odd. Authors in these fields ask us to pay attention to our intricately connected material world as a mode of everyday being as well as a method of imaginative and critical practice. Discussing such work in a virtual setting feels ironic, although high tech connectivity provides both thought-provoking metaphor and contrast for world ecology; and clever observations on the watery rhetoric through which we describe the world wide web can précis more meaningful discussion. Nonetheless, focusing on a screen takes us away from contemplating our ‘tentacular’ connections within our immediate material worlds (Haraway 2016).

As I write and teach, I occasionally glance away from my screen to look at a tiny brown spider on the wall, romantically connecting my suburban room with Haraway’s epic sense of the many-limbed and microscopic. Or I consider what it can mean for me to gaze at our black cat looking at me looking at her. With Michel de Montaigne, I wonder who is really in control here (1877[1580]). We have asserted our ownership by naming her, but surely she has shaped and domesticated us to her needs and whims. With Jacques Derrida, I strive to see the cat, not as the categorical animal ‘other’ symbolising the violence of Western intellectual and industrial history, but as herself; specific, present, individuated, and familiar in ways that are mysterious but more than a mere shared capacity to experience pain (2008). These days, these indoor creatures—mammalian and insect, squashable and clawed—are my kin and critter—my in-common—more fully than ever. There is another irony; that being inside more than usual has brought so many of us closer to these creaturely commons. I oscillate between their occurrence as a scene of self-conscious academic critique and a matter of everyday life. I think and feel. And I think and feel that these new in-commons—indoors and outdoors, background and foreground—describe a new normal for both life and critique.
